# Magnetically recyclable CoFe_2_O_4_/ZnO nanocatalysts for the efficient catalytic degradation of Acid Blue 113 under ambient conditions

**DOI:** 10.1039/d0ra00082e

**Published:** 2020-04-25

**Authors:** S. Krishna, P. Sathishkumar, N. Pugazhenthiran, Kiros Guesh, R. V. Mangalaraja, S. Kumaran, M. A. Gracia-Pinilla, S. Anandan

**Affiliations:** Department of Chemistry, Periyar Maniammai Institute of Science & Technology Vallam Thanjavur 613403 Tamil Nadu India; Department of Chemistry, Aksum University Axum 1010 Ethiopia sathishpanner2001@gmail.com; Laboratorio de Tecnologías Limpias, Facultad de Ingeniería, Universidad Católica de la Santísima Concepción Alonso de Ribera 2850 Concepción Chile; Advanced Ceramics and Nanotechnology Laboratory, Department of Materials Engineering, Faculty of Engineering, University of Concepcion Concepcion 4070409 Chile; Department of Biotechnology, Periyar Maniammai Institute of Science & Technology Vallam Thanjavur 613 403 Tamil Nadu India; Universidad Autonoma de Nuevo Leon, Facultad de Ciencias Físico-Matematicas, Av. Universidad, Cd. Universitaria San Nicolas de los Garza N.L. Mexico; Nanomaterials and Solar Energy Conversion Lab, Department of Chemistry, National Institute of Technology Trichy 620015 India

## Abstract

CoFe_2_O_4_/ZnO magnetic nanocatalysts were synthesized using a low-frequency ultrasound-assisted technique to enhance the optical, morphological, magnetic and catalytic properties of ZnO. The as-synthesized nanocatalysts were characterized by XRD, Raman, TEM, DR-UV-Vis and VSM analyses in order to confirm the expected modifications of the resulting nanocatalysts. The Raman spectral analysis revealed substitutional Zn^2+^ in the CoFe_2_O_4_/ZnO nanocatalyst. The as-synthesized material was tested for its catalytic activity in the degradation of Acid Blue (AB113), a known textile pollutant. The CoFe_2_O_4_ and CoFe_2_O_4_/ZnO nanocatalysts revealed the efficient catalytic degradation of AB113 in ambient conditions. The nanocatalyst dosage and the initial concentration of AB113 were varied by fixing one parameter as constant in order to determine the maximum catalytic efficiency with the minimum catalyst loading for AB113 degradation. The CoFe_2_O_4_/ZnO nanocatalyst demonstrated 10-fold enhanced mineralization of AB113 compared to the individual bare nanocatalysts, which could be achieved within 3 hours of catalytic degradation of AB113. The magnetic CoFe_2_O_4_/ZnO nanocatalyst was found to be stable for six consecutive recycles of AB113 degradation, which indicates that the catalytic efficiency of the nanocatalyst was retained after various numbers of cycles.

## Abstract 2

CoFe_2_O_4_/ZnO magnetic nanocatalysts were synthesized using a low-frequency ultrasound-assisted technique to enhance the optical, morphological, magnetic and catalytic properties of ZnO.

## Graphical abstract

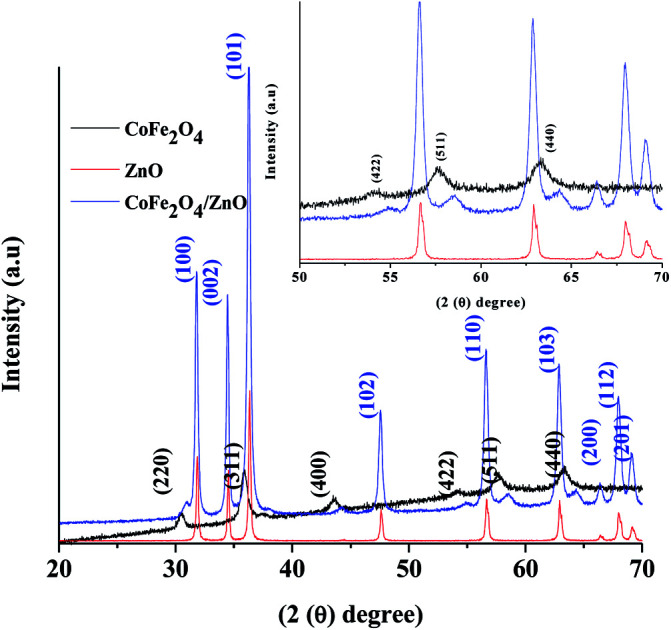

## 1 Introduction

The degradation of environmental pollutants in ambient environmental conditions is a prodigious challenge for researchers because it avoids the drawbacks associated with conventional degradation techniques and reduces the capital investment required for the installation of treatment plants. In addition, the catalytic conversion of environmental contaminants into non-hazardous end-products in the absence of external energy is a great challenge for researchers. In the last five decades of research in the field of environmental contaminants, several treatment techniques based on the utilization of various reagents, chemicals, and energy sources have been reported. However, the reported methodologies involving the application of external energy sources are expensive.^1–3^ Moreover, unsafe disposal of the catalysts utilized in the degradation processes creates secondary pollution.^4,5^ Another difficulty associated with the reported technologies is incomplete degradation of the pollutants, which also creates secondary pollution in the environment. In addition, the prolonged treatment time and the cost required for the degradation of environmental pollutants prevent the commercialization of these technologies.^1^ The unique properties associated with nanocatalysts may address the issues related to heterogeneous catalysis. The introduction of magnetic properties to non-magnetic materials enables recovery and re-usage of the nanocatalysts which are utilized in the degradation of environmental contaminants.^5–10^

Nanocatalysts with magnetic properties can be utilized as catalysts in ambient conditions, and the magnetic properties of the nanocatalysts can enhance the possibility of reutilization in addition to decreasing the cost required for the overall processes.^11–13^ The electronic charges created during the catalytic processes tend to generate reactive oxygen species (ROS). The ROS rapidly degrade the organic pollutants present in the aqueous medium into non-hazardous products. The recombination of these electronic charges can be precluded by the surface defects, surface functional groups, sizes, and shapes of the nanocatalysts applied in the nanocatalysis processes. Dimensional anisotropy also plays a crucial role in deciding the efficacy of nanocatalysts.^14,15^

Reports of the degradation of environmental contaminants in ambient conditions are rare and limited. BaFeO_3_ nanocatalysts degraded 50% of the initial methyl orange dye concentration in dark conditions with a prolonged reaction time (5 days).^16^ The replacement of barium by strontium enhanced the degradation of acid orange by shortening the reaction time by 8 to 60 minutes.^17^ Later, CeO_2_ was added to the SrFeO_3_ nanocatalyst to degrade dye effluents within a shorter time duration under dark catalysis conditions.^18^ Similarly, LaCuO_3_, LaTiCuO_3_, CaSrCuO_3_, BaSrCoCuO_3_ and Cu_2_O/ZnO nanocomposites were found to be suitable nanocatalysts for the degradation of environmental contaminants in dark/ambient conditions.^12,13,19–23^ Polyaniline (PANI)-based catalysts with magnetic nanoparticles demonstrated enhanced catalytic efficiency and can also be separated by the application of an external magnet.^24,25^ The introduction of Zn^2+^ into NiFe_2_O_4_ enhanced the catalytic and magnetic properties of the resulting nanocatalysts.^26^ The reported dark/ambient nanocatalysts degraded various environmental contaminants by Fenton-like reactions, and a synergetic effect was achieved when FeO_3_ or CuO was attached to other metal oxides.^22,23^

Considering the available reported literature in the field of ambient catalytic efficiency for various nanocatalysts, in the present work, CoFe_2_O_4_/ZnO hybrid nanocomposites were synthesized using an economically viable low-frequency ultrasound-assisted method to enhance the catalytic and magnetic properties of the resulting nanocatalysts. The catalytic efficiency was monitored by following the degradation kinetics of the model pollutant Acid Blue 113. The spinel CoFe_2_O_4_ nanoparticles were expected to prevent the leaching of each metal constituent by the formation of Co–Fe metal bonds because the release of Co^2+^ is harmful to the environment.^5,10^ The formation of new hybrid energy levels in the nanocatalysts was anticipated to enhance the catalytic and magnetic efficiency of the resulting nanocatalysts.

## 2 Experimental

### 2.1 Materials

Cobalt nitrate (Co(NO_3_)_2_·6H_2_O), ferric nitrate (Fe(NO_3_)_3_·9H_2_O) and zinc oxide (ZnO) were purchased from Sigma-Aldrich and were used as starting materials for the preparation of the CoFe_2_O_4_ and CoFe_2_O_4_/ZnO nanocatalysts. Acid Blue 113 (diazo dye, C_32_H_21_N_5_Na_2_O_6_S_2_; C.I. 26360) was received from Sigma-Aldrich and used without further purification. Unless otherwise specified, all reagents used were of analytical grade, and the solutions were prepared using double distilled water.

### 2.2 Preparation of the nanocatalysts

The cobalt nitrate and ferric nitrate precursor solutions with calculated amounts were prepared separately and mixed under vigorous stirring. To the metal nitrate mixture, 2 g of previously synthesized ZnO nanopowder was added in small portions with magnetic stirring; simultaneously, the suspension was irradiated with ultrasound (40 kHz ultrasonic cleaner, Power Sonic 400 series). To a suspension of the ZnO-metal precursors, 50 ml of 2 N NaOH was added dropwise with ultrasound irradiation and stirring. The ultrasonic irradiation was continued for one hour with circulation of the water in order to maintain room temperature. After the ultrasonic irradiation, the heterogeneous suspension was stirred continuously at room temperature for two hours. The nanocatalysts were collected by filtration (0.45 μm nylon filter membranes) and washed with double-distilled water until the pH of the washing solution reached 7. The solids were dried at 100 °C in a hot air oven for 12 h, followed by calcination at 400 °C for 5 h to afford the pure CoFe_2_O_4_/ZnO nanocatalyst. A similar procedure was adopted for the synthesis of a CoFe_2_O_4_ nanocatalyst without ZnO for comparison. A ZnO nanocatalyst was prepared using a similar process in the absence of Co–Fe metal precursor solution.

### 2.3 Characterization techniques

The particle sizes of the prepared nanoparticles were calculated from the X-ray diffraction data (Philips PW1710 diffractometer, CuK_α_ radiation, Holland) using the Scherrer equation. The surface morphologies, particle sizes, and various contours of the nanocatalyst powders were analyzed by transmission electron microscopy (FEI TITAN G^2^ 80-300) operated at 300 keV. Diffuse reflectance UV-Vis spectra of the nanocatalysts were recorded using a Shimadzu 2550 spectrophotometer equipped with an integrating sphere accessory using BaSO_4_ as the reference material. Raman spectra were recorded using an inVia Raman spectrometer (Renishaw, UK) operating at a wavelength at 785 nm with a resolution of 1 cm^−1^. The magnetization measurements were performed with a physical property measurement system (Quantum Design PPMS-II) under ambient conditions. The total organic carbon (TOC) for all the samples was analyzed by direct injection of the filtered sample solution into a TOC analyzer (Shimadzu TOC-VCPH model). Prior to the TOC analysis, the instrument was calibrated with potassium hydrogen phthalate. TOC_0_ is the TOC measured after the equilibrium adsorption of the dye on the nanocatalyst surface, and the TOCs obtained at various irradiation times are denoted as TOC_*t*_.

### 2.4 Evaluation of catalytic efficiency

The catalytic degradation of Acid Blue 113 (AB113) was evaluated by adding a calculated amount of nanocatalyst to a freshly prepared dye solution with the required concentration in ambient environmental conditions at the natural solution pH (∼6.0). The nanocatalyst was added, and the time was immediately noted as “zero” for catalytic degradation. Aliquots were withdrawn at various time intervals and analyzed by the UV-Vis spectrophotometer in order to measure the degradation of AB113 (*λ*_max_ = 567 nm). The catalytic degradation of AB113 was studied from 1 × 10^−6^ M to 1 × 10^−4^ M concentrations of AB113, and the nanocatalyst dosage was varied from 0.2 to 5.0 g L^−1^ with a fixed initial AB113 concentration.

## 3 Results and discussion

### 3.1 Characterization of the nanocatalysts

**Fig. 1:**
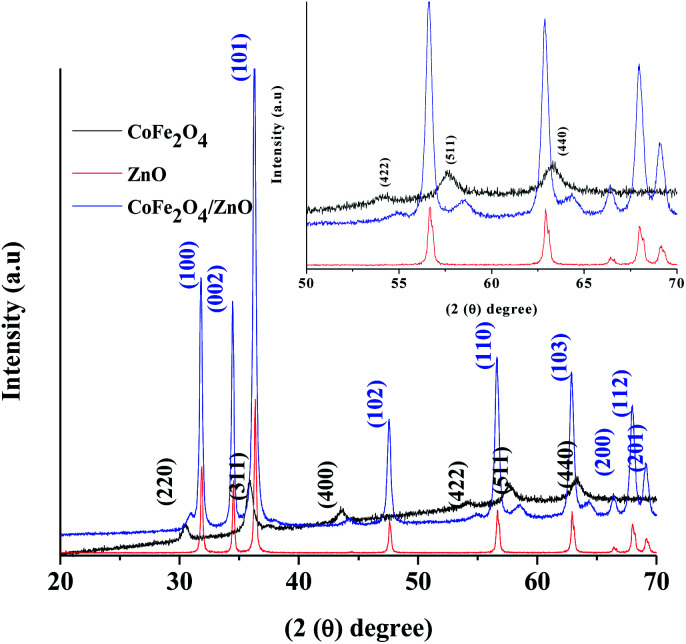
X-ray diffraction patterns of the various nanocatalysts synthesized by the ultrasound-assisted technique. The insert shows the magnified XRD patterns of the nanocatalysts.

The X-ray diffraction patterns of the various nanocatalysts synthesized by the ultrasound-assisted technique are shown in Fig. 1. The XRD patterns confirm that CoFe_2_O_4_ (black line) exists in the spinel crystal structures according to JCPDS card no. 22-1086, whereas the ZnO XRD pattern (red line) confirms the existence of wurtzite crystal structure, corresponding to JCPDS card no. 36-1451. The wurtzite structure is retained even after the modification with CoFe_2_O_4_, as evidenced in Fig. 1 (blue line), and the CoFe_2_O_4_ peaks appeared in the pattern of the modified CoFe_2_O_4_/ZnO; this indicates that the spinel-structured CoFe_2_O_4_ exists along with the wurtzite structure of ZnO. Fig. 1 (insert) displays the magnified XRD patterns of the synthesized nanocatalysts at 2*θ* (50–70°); the crystal planes (422), (511) and (440) corresponding to CoFe_2_O_4_ significantly shifted from their original positions in the resulting CoFe_2_O_4_/ZnO nanocatalyst. This confirms that the maximum amount of CoFe_2_O_4_ nanocatalyst exists on the surface of the CoFe_2_O_4_/ZnO nanocatalyst. However, a small portion of Zn^2+^ may have entered the crystal structure of CoFe_2_O_4_ during the calcination process, which cannot be identified in the XRD patterns. No other peaks were identified in the XRD patterns (Fig. 1) of the various nanocatalysts, indicating that highly pure nanocatalysts were obtained by the synthetic procedure.

**Fig. 2:**
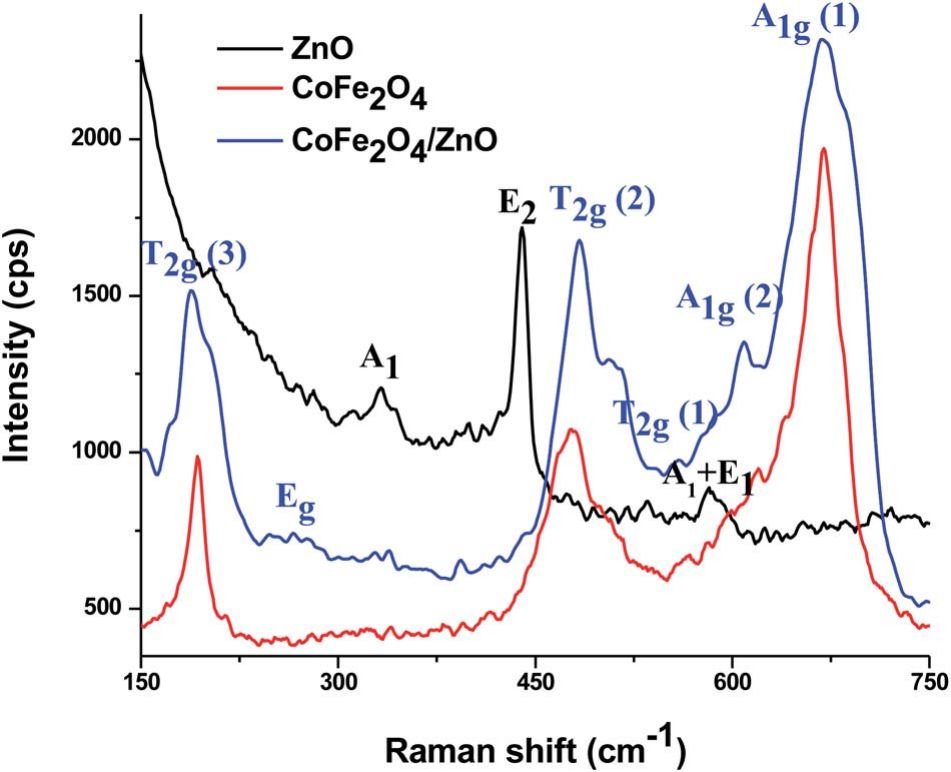
Raman spectra of the various nanocatalysts recorded at room temperature.

Raman spectra were recorded at room temperature for the ZnO, CoFe_2_O_4_ and CoFe_2_O_4_/ZnO nanocatalysts to determine whether Zn^2+^ diffused into the crystal structure of CoFe_2_O_4_ (Fig. 2) because the CoFe_2_O_4_/ZnO nanocatalyst exhibits catalytic degradation of Acid Blue 113 in ambient conditions, which kindled our curiosity to find the reason for this catalytic activity. The bare ZnO shows Raman active modes corresponding to 330, 437 and 574 cm^−1^, which can be attributed to the wurtzite crystal structure belonging to the space group C_6*v*_^4^. The Raman peak at ∼330 cm^−1^ can be assigned to the second-order vibrational mode. The strongest Raman peak appearing at ∼437 cm^−1^ indicates E_2_ mode, whereas the Raman peak observed at ∼574 cm^−1^ denotes a mixture of A_1_ and E_1_ modes.^27^ In addition, the peak at 574 cm^−1^ demonstrates E_1_ symmetry with the longitudinal-optical phonon mode, which arises from the oxygen vacancies, free charge carriers and interstitial defects of ZnO.

For the spinel structure of CoFe_2_O_4_, which belongs to the O_h_^7^ (*Fd*3*m*) space group, Raman-active peaks are expected for the A_1g_, E_g_ and 3T_2g_ modes. The Raman active peaks at ∼189, 270, 480, 561, 605 and 675 cm^−1^ can be seen in Fig. 2 for the CoFe_2_O_4_ and CoFe_2_O_4_/ZnO nanoparticles. The Raman peak originating at ∼675 cm^−1^ (A_1g_ (1)) along with the peak at ∼605 cm^−1^ (A_1g_ (2)) designate M–O stretching vibrations in tetrahedral sites. The other Raman active peaks (∼189, 270, 480 and 561 cm^−1^) can be assigned to the T_2_g and E_g_ Raman active modes, which demonstrates the spinel structure vibrations. Additionally, the symmetric bending and asymmetric stretching of oxygen ions are associated with the A_1g_ and E_g_ modes, respectively, whereas the T_2g_ mode is associated with the asymmetric stretching of oxygen.^28^ A_1g_ symmetry related to the tetrahedral and octahedral crystal lattice sites at 650 to 710 cm^−1^ was reported for inverse spinel ferrites. However, A_1g_ symmetry at 600–650 cm^−1^ was reported for normal spinel ferrites.^29,30^ Due to the enhanced intensity observed for CoFe_2_O_4_/ZnO compared to the bare CoFe_2_O_4_, it can be presumed that Zn^2+^ ions substitutionally entered the CoFe_2_O_4_ crystal structure.

**Fig. 3:**
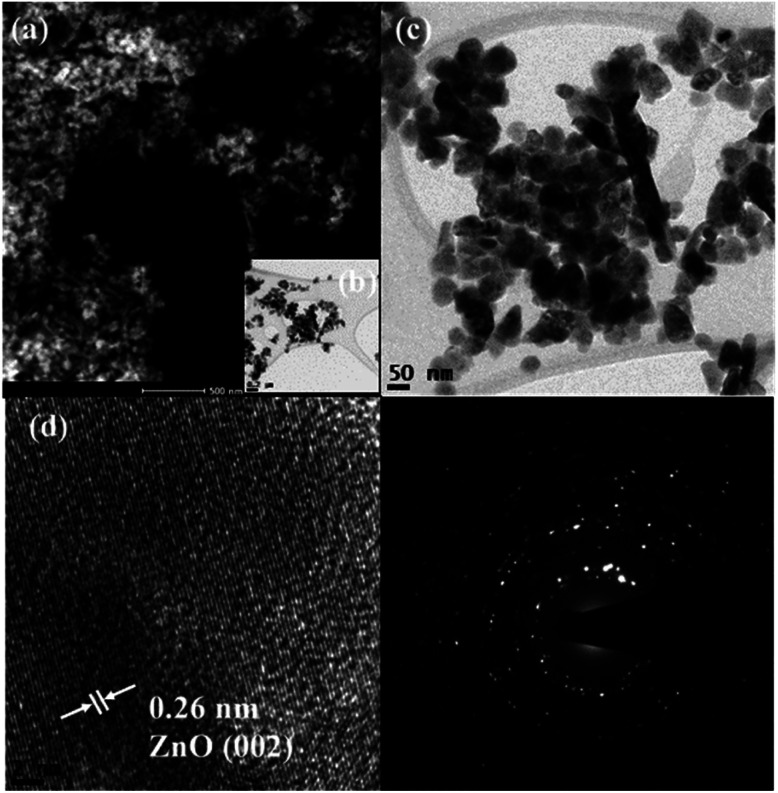
Representative STEM-HAADF image (a), TEM images (b–d) and SAED pattern (e) of the ZnO nanocatalyst.

**Fig. 4:**
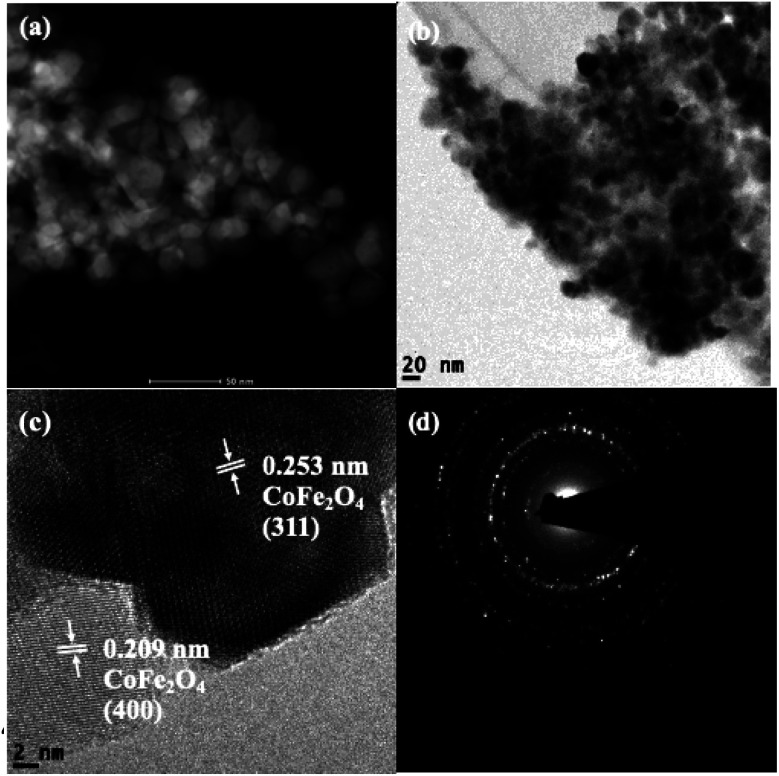
Representative STEM-HAADF image (a), TEM images (b and c) and SAED pattern (d) of the CoFe_2_O_4_ nanocatalyst.

Scanning transmission electron microscopy coupled with high-angle annular dark-field (STEM-HAADF) images demonstrated the formation of nanorods and nanoparticles of ZnO (Fig. 3a). The nanorods were formed during the preparation of the ZnO nanocatalyst. However, destruction of the nanorods during the ultrasound irradiation led to the formation of nanoparticles. The STEM-HAADF observations were well supported by the TEM analysis, which shows the formation of nanorods and nanoparticles of ZnO (Fig. 3b). The diameter of the nanorods was 30–40 nm, whereas lengths of the nanorods of a few micrometers can be observed from Fig. 3a–c. An average size of the ZnO nanoparticles of 30 nm was evidenced from the TEM (Fig. 3c), which is in good agreement with the particle size calculated from the XRD pattern. In addition, coarsening was observed in the resulting nanoparticles due to the calcination and ultrasound (Fig. 3c). The lattice fringe distance calculated from Fig. 3d designates the formation of ZnO (002) crystal planes, and the corresponding SAED pattern (Fig. 3e) displays the multi-crystalline nature of the synthesized ZnO. The STEM-HAADF and TEM images exhibit the presence of CoFe_2_O_4_ in the nanoparticle structures (Fig. 4a and b) with an average size of ∼15 nm. The HRTEM analysis indicates the formation of various crystal planes of CoFe_2_O_4_ nanoparticles. However, the (311) and (400) crystal planes were predominantly formed during the preparation (Fig. 4c). Lattice diffusion and overlapping of various crystal planes can also observed in Fig. 4c, which indicates the formation of multilayered crystal structures of the CoFe_2_O_4_ nanoparticles. The SAED pattern also confirms the presence of multi-crystallinity in the synthesized CoFe_2_O_4_ nanoparticles (Fig. 4d).

**Fig. 5:**
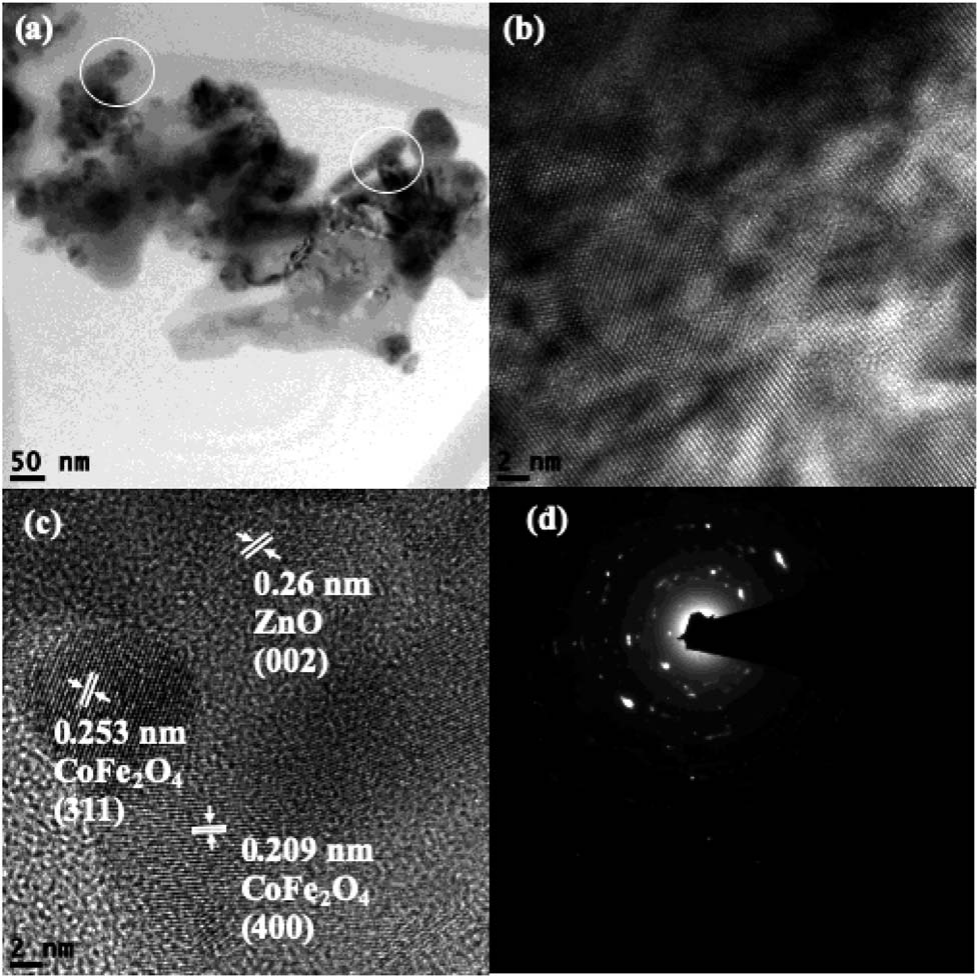
TEM images of the CoFe_2_O_4_/ZnO nanocatalyst (a–c) and the corresponding SAED pattern (d).

The TEM analysis of CoFe_2_O_4_/ZnO shows that clustering of the nanocatalysts occurred during their preparation and post-treatment. Nanorods were perceived in the ZnO-supported CoFe_2_O_4_ nanocatalyst (circled in Fig. 5a), confirming that the nanorods were initially formed during the synthesis. However, the formation of CoFe_2_O_4_ along with ZnO led to the formation of nanoparticles and nanorods, which is evidenced in Fig. 5a. In addition, the distribution of black patches (CoFe_2_O_4_) over the gray surface (ZnO) indicates the predominant distribution of CoFe_2_O_4_ on the surface of ZnO. The HRTEM images (Fig. 5b and c) further authenticate the formation of a multilayered CoFe_2_O_4_/ZnO nanocatalyst by the low-frequency ultrasound process. The lattice fringe distance calculated from the TEM analysis corroborates the formation of ZnO (002) and CoFe_2_O_4_ ((311), (400)) crystal planes. The SAED multi ring pattern also supported the formation of highly pure CoFe_2_O_4_/ZnO nanocatalyst. In other words, no impurities were found in the synthesized CoFe_2_O_4_/ZnO nanocatalyst. However, the substitutional entry of Zn^2+^ into the CoFe_2_O_4_ crystal lattice was not identified from the TEM analysis.

**Fig. 6:**
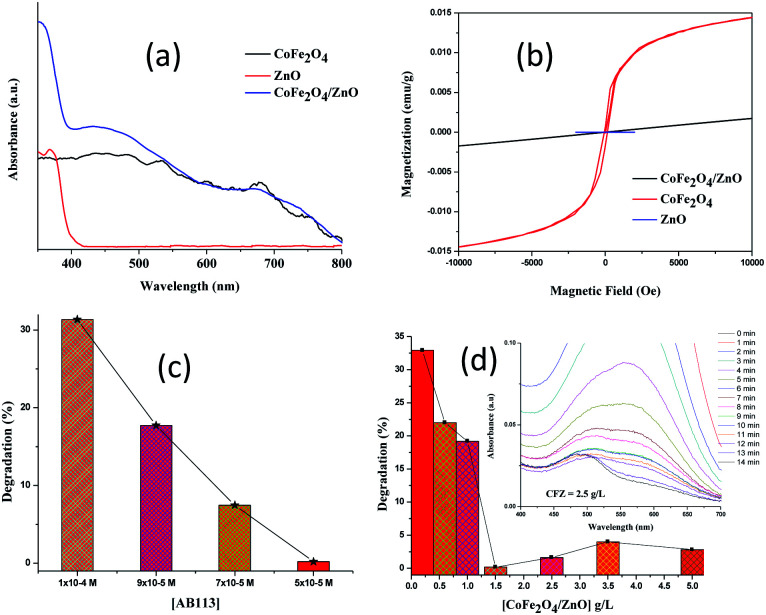
DR-UV-Vis diffuse reflectance spectra (a) and magnetic studies (b) of the various nanocatalysts synthesized using low-frequency ultrasound; percentages of degradation of Acid Blue 113 at various concentrations in the presence of a fixed CoFe_2_O_4_/ZnO nanocatalyst dosage (1.5 g L^−1^) (c); and percentages of degradation of Acid Blue 113 [5 × 10^−5^ M] in the presence of various dosages of CoFe_2_O_4_/ZnO (d). The insert shows the observed characteristic changes in the UV-Vis spectra of AB113 degradation.

The optical properties of the synthesized nanocatalysts were studied using diffuse reflectance (DR) UV-vis spectral analysis (Fig. 6a). The absorption band shifted from ∼390 nm for the CoFe_2_O_4_/ZnO nanocatalyst compared to bare ZnO, which indicates that the optical properties of ZnO were significantly modified in the presence of CoFe_2_O_4_. The formation of new energy levels can be attributed to the observed red-shift in the CoFe_2_O_4_/ZnO nanocatalyst from ∼390 nm in pure ZnO. CoFe_2_O_4_ is categorized as a low-band gap semiconductor; therefore, it does not show any significant peaks in the visible range of the spectrum. However, the appearance of various humps ranging from 500 nm to 750 nm in the DR UV-vis spectrum of CoFe_2_O_4_ validates the existence of oxides of Co and Fe cations. The absence of CoFe_2_O_4_ humps in the spectrum of the CoFe_2_O_4_/ZnO nanocatalyst endorses that the CoFe_2_O_4_ was surmounted by the ZnO nanocatalysts. It is expected that in addition to the optical properties, the magnetic properties were modified due to the covered CoFe_2_O_4_ nanoparticles.

The magnetic properties of the synthesized nanocatalysts were studied at room temperature, as shown in Fig. 6b. As expected, the ZnO nanoparticles do not show any significant magnetic properties. The CoFe_2_O_4_ nanoparticles demonstrated characteristic magnetic behavior with a large hysteresis loop. The engineering of non-magnetic nanomaterials with magnetic nanoparticles tends to modify the magnetic properties of the resulting nanocatalysts. CoFe_2_O_4_/ZnO exhibits lower magnetic strength compared to CoFe_2_O_4_, whereas its magnetic field strength perceptibly increased compared to bare ZnO, as evidenced in Fig. 6b. The magnetization of CoFe_2_O_4_/ZnO strongly depends on the Zn^2+^ content and its substitution in the crystal lattices of the resulting nanocatalyst.^31^ It can be concluded from the magnetic studies that a stronger external magnetic field is required to separate the CoFe_2_O_4_/ZnO nanocatalyst compared to the CoFe_2_O_4_ nanoparticles for the recovery and re-utilization of the magnetic nanocatalysts.

### 3.2 Catalytic degradation of AB113

The adsorption of AB113 was found to be prominent for all the AB113 concentrations within the first minute of the reaction, which gave a strong indication that the dye molecules adhered immediately after the addition of nanocatalyst. Complete decolorization of AB113 was achieved within 3 minutes of magnetic stirring at a low concentration of dye (1× 10^−6^ M to 3 × 10^−5^ M). The rapid degradation of AB113 was attained due to the generation of an enhanced number of free radicals immediately after the addition of the nanocatalyst. However, with increasing AB113 concentration, an extended reaction time was required to achieve complete decolorization. With an AB113 concentration of 5 × 10^−5^ M, the decolorization of the dye required 15 minutes, whereas with 7 × 10^−5^ M and 9 × 10^−5^ M concentrations, 27 and 46 minutes were needed, respectively. The catalytic degradation of AB113 at various concentrations with a fixed amount of nanocatalyst (1.5 g L^−1^) is depicted in Fig. 6c to compare the degradation efficiencies within 15 minutes of reaction time. The increase in the concentration of AB113 with a fixed nanocatalyst dosage (1.5 g L^−1^) renders the competition between the dye molecules for the adoption. The continuous decrease observed in *λ*_max_ during UV-Vis spectral analysis indicates that the dye molecules adsorbed initially tend to degrade and be released from the nanocatalyst surface. Later, the proximate dye molecules in the solution are adsorbed for degradation. However, upon further increase in the dye concentration (1 × 10^−4^ M), ∼2 hours were required for the decolorization under ambient conditions.

The AB113 concentration was fixed at 5 × 10^−5^ M and the nanocatalyst dosage was varied (0.2 g L^−1^ to 5 g L^−1^) to attain the maximum degradation efficiency. The maximum adsorption of dye molecules (∼50%) was initially observed for all the studied concentrations of the CoFe_2_O_4_/ZnO nanocatalyst, which indicates that the binding of AB113 on the nanocatalyst surface is very high. At a low concentration of nanocatalyst (0.2–0.6 g L^−1^), the *λ*_max_ of AB113 was found to be inconsistent because small portions of the adsorbed dye molecules were released instead of being degraded during the nanocatalysis. However, ∼65% AB113 degradation was achieved with the minimum dosage of the CoFe_2_O_4_/ZnO nanocatalysts within 15 minutes of reaction (Fig. 6d).

An increase in the loading of CoFe_2_O_4_/ZnO above 0.6 g L^−1^ demonstrated consistent UV-Vis spectra for AB113; this indicates that the adsorbed dye molecules were not released from the nanocatalysts and persisted further. Fig. 6d shows the percentage degradation of AB113 degradation at higher catalyst dosages [1.5–5.0 g L^−1^]. The maximum decolorization of AB113 was achieved within 15 minutes of magnetic stirring at ambient conditions {[AB113] = 5 × 10^−5^ M and [CFZ] = 1.5 g L^−1^}. The adsorption followed by the immediate generation of reactive oxygen species (ROS) tends to rapidly decolorize AB113. The prompt generation of ROS can be achieved due to the multilayer structures of the synthesized nanocatalysts. The generation of electronic charges (electron–hole) is enhanced, and they react in turn with H_2_O to form an augmented number of ROS for the rapid decolorization of AB113 within a short reaction time.^12,13,32,33^ The loading of [CoFe_2_O_4_/ZnO] = 2.5 g L^−1^ exhibits intermediate formation after 12 minutes of reaction (insert of Fig. 6d). This confirms that the degradation of AB113 was initiated in addition to its decolorization, and the observed increase in the intensity of the peak at ∼494 nm confirms the formation of byproducts of AB113. Further increase in the reaction time increased the degradation of AB113 and its byproducts. A similar trend was observed when the catalyst dosage was increased to 3.5 g L^−1^. In addition, when the concentration of catalyst was increased to 5.0 g L^−1^, the characteristic absorbance of AB113 immediately vanished and the appearance of new peaks with increasing concentration and reaction time was noted. This confirms that an enhanced number of ROS formed compared to the low catalytic dosage, leading to the rapid degradation of AB113.

**Fig. 7:**
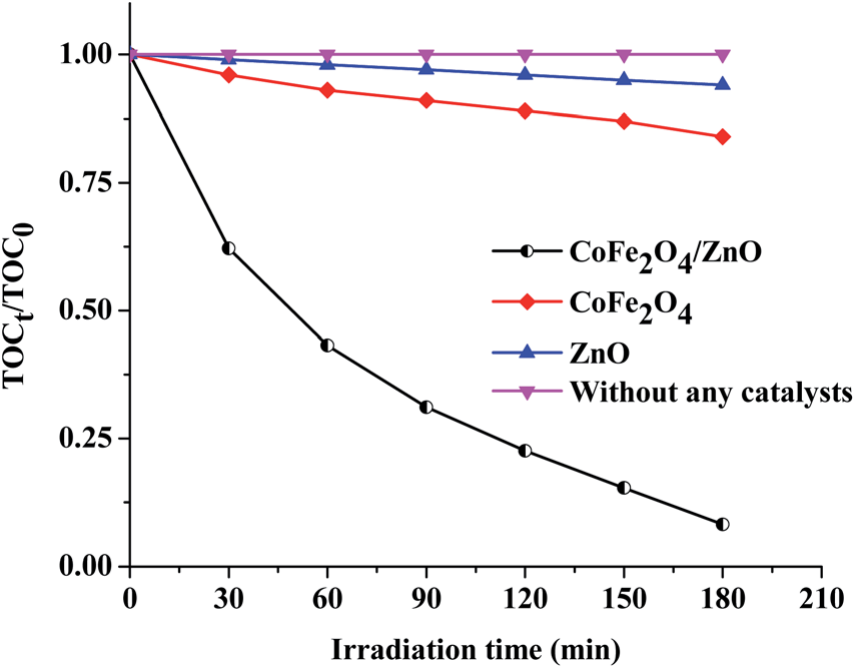
Comparison of the mineralization of AB113 in the absence and presence of various nanocatalysts under ambient environmental conditions at fixed initial concentrations of nanocatalyst [CoFe_2_O_4_, ZnO and CoFe_2_O_4_/ZnO] = 1.5 g L^−1^ and [AB113] = 5 × 10^−5^ M.

The optimized concentrations of the nanocatalysts (ZnO, CoFe_2_O_4_ and CoFe_2_O_4_/ZnO, 1.5 g L^−1^) and of AB113 (5 × 10^−5^ M) were fixed as the initial concentrations to study the mineralization of AB113. The obtained mineralization results (Fig. 7) suggest that AB113 cannot be mineralized in ambient environmental conditions (absence of nanocatalyst). The ZnO nanocatalysts exhibited ∼6% total organic carbon (TOC) reduction compared to the initial TOC (TOC_0_) and the TOC obtained after 180 minutes (TOC_180_). The optical band gap of ZnO is ∼3.32 eV, which cannot generate electronic charges in the ambient environmental conditions; however, the intrinsic, crystal and surface defects associated with the wurtzite crystal structure tend to exhibit very low light absorption. Due to their low band gap, the CoFe_2_O_4_ nanoparticles demonstrated enhanced TOC removal (∼15%) compared to the TOC reduction in the presence of ZnO. The obtained TOC reduction can be attributed to the generation of electronic charges under ambient environmental conditions; however, the recombination of electronic charges is higher than the quantum efficiency of the CoFe_2_O_4_ nanocatalyst because of the low band gap (∼1.1 eV). The CoFe_2_O_4_/ZnO nanocatalyst showed approximately 10-fold enhanced mineralization, which can be achieved within 3 hours of the catalytic degradation of AB113 compared to its individual bare nanocatalysts. The obtained enhanced TOC reduction can be explained based on the enhanced generation of ROS under the ambient environmental conditions. It can be inferred from the mineralization studies (Fig. 7) that CoFe_2_O_4_/ZnO demonstrated enhanced mineralization of AB113 compared to the CoFe_2_O_4_ and ZnO nanocatalysts during the progress of the catalytic degradation of AB113 under ambient environmental conditions.

**Fig. 8:**
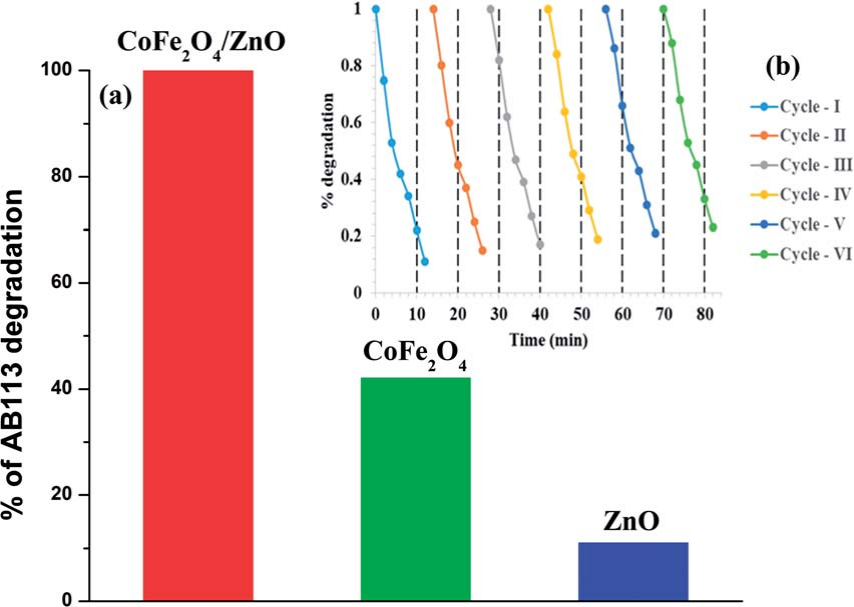
Comparison of the catalytic efficiency of CoFe_2_O_4_/ZnO and its bare nanocatalysts under ambient conditions within 15 minutes of catalytic degradation (a) and catalytic degradation of AB113 in the presence of the magnetically recyclable CoFe_2_O_4_/ZnO nanocatalyst for six cycles (b). The concentrations were as follows: [AB113] = 5 × 10^−5^ M and [CoFe_2_O_4_/ZnO] = 2.5 g L^−1^.

In order to compare the catalytic efficiency of the synthesized nanocatalysts, the concentrations of AB113 and the nanocatalyst were fixed as 5 × 10^−5^ M and 2.5 g L^−1^, respectively, and the results are shown in Fig. 8a. The catalytic efficiency of ZnO was found to be very low or negligible compared to the catalytic efficiencies of the CoFe_2_O_4_ and CoFe_2_O_4_/ZnO nanocatalysts. 2.5-fold enhanced catalytic efficiency was achieved for the CoFe_2_O_4_/ZnO nanocatalyst compared to the catalytic efficiency achieved for the bare CoFe_2_O_4_ nanocatalyst within a short time duration (15 minutes) of the catalysis. The degradation of AB113 may have been enhanced due to the substitutional occupation of Zn^2+^ ions in the CoFe_2_O_4_/ZnO nanocatalyst because this substitution alters the crystallographic and magnetic properties of the resulting nanocatalyst, which is evidenced by the Raman spectral analysis (Fig. 2) and magnetization hysteresis loops (Fig. 6b). However, the bare CoFe_2_O_4_ nanoparticles exhibit significant catalytic efficiency, which can be attributed to their low band-gap. The presence of Zn^2+^ ions at the interstitial positions of the CoFe_2_O_4_/ZnO nanocatalyst can act as a trap for the electronic charges created during the ambient catalysis conditions, which further enhances the generation of ROS within the catalytic microenvironment. Therefore, catalytic degradation of AB113 can be attained within 15 minutes of the catalytic reaction. However, further mechanistic analysis is needed; this will be published soon. Recycling experiments were performed to determine the stability and efficiency of the CoFe_2_O_4_/ZnO nanocatalyst. The nanocatalyst was separated from the catalytic microenvironment by the application of an external magnet after the degradation of AB113. The nanocatalyst was washed with distilled water and dried at room temperature before each cycle of the experiment (Fig. 8b). The nanocatalyst exhibited almost the same efficiency for six cycles, which further confirmed the stability of the nanocatalyst. However, a small portion of substrate adsorption was observed in each cycle, which indicates that ∼90% catalytic efficiency was retained even after six consecutive cycles of catalytic degradation.

## 4 Conclusion

In this study, CoFe_2_O_4_/ZnO, CoFe_2_O_4_ and ZnO nanocatalysts were synthesized by a low-frequency ultrasound-assisted technique in order to enhance the magnetic and catalytic behavior of the resulting nanocatalysts. The XRD, TEM and DR UV-Vis spectral analyses confirmed that the ZnO and CoFe_2_O_4_ nanoparticles retained their crystal structures even after the modification of the nanocatalysts, and the CoFe_2_O_4_ crystal planes were found to exist at the ZnO surface; this gave the first indication that the expected modifications were achieved. However, the Raman analysis revealed that Zn^2+^ ions had substitutionally entered the crystal structure of CoFe_2_O_4_, which endows the CoFe_2_O_4_/ZnO nanocatalyst with ambient catalytic efficiency. The characterization techniques revealed that the low-frequency ultrasound created the expected modifications in the resulting nanocatalysts. The modification of CoFe_2_O_4_ endows the CoFe_2_O_4_/ZnO nanocatalyst with magnetic properties. However, the magnetic strength was found to be reduced compared to that of bare CoFe_2_O_4_ due to the engineering of non-magnetic semiconductors into the magnetic nanoparticles. The magnetic properties along with the ambient catalytic efficiency of the CoFe_2_O_4_/ZnO nanocatalyst can avoid the primary and secondary pollution associated with the direct/indirect disposal of environmental contaminants and/nanocatalysts into the environment. The mineralization studies indicated that AB113 can be mineralized within 3 hours of catalytic degradation under ambient environmental conditions and demonstrated the multi-fold enhanced mineralization of the CoFe_2_O_4_/ZnO nanocatalyst compared with its individual parts. In addition, the recycling efficiency, morphological stability, and catalytic efficiency of the magnetic nanoparticles were demonstrated for the CoFe_2_O_4_/ZnO nanocatalyst.

## Conflicts of interest

There are no conflicts of interest to declare.

## Supplementary Material

## References

[cit1] Mahamuni N. N., Adewuyi Y. G. (2010). Ultrason. Sonochem..

[cit2] Gogate P. R., Mujumdar S., Pandit A. B. (2002). Ind. Eng. Chem. Res..

[cit3] Khokhawala I. M., Gogate P. R. (2010). Ultrason. Sonochem..

[cit4] Han Q., Yang S., Yang X., Shao X., Niu R., Wang L. (2012). Prog. Chem..

[cit5] Sathishkumar P., Mangalaraja R. V., Anandan S., Ashokkumar M. (2013). Chem. Eng. J..

[cit6] Wilson A., Mishra S. R., Gupta R., Ghosh K. (2012). J. Magn. Magn. Mater..

[cit7] Zeng Q., Jiang D., Yang S. (2016). RSC Adv..

[cit8] Zhang G., Xu W., Li Z., Hu W., Wang Y. (2009). J. Magn. Magn. Mater..

[cit9] Zheng J., Song X., Liu X., Chen W., Li Y., Guo J. (2012). Mater. Lett..

[cit10] Sathishkumar P., Pugazhenthiran N., Mangalaraja R. V., Asiri A. M., Anandan S. (2013). J. Hazard. Mater..

[cit11] Wu J. M., Wen W. (2010). Environ. Sci. Technol..

[cit12] Chen H., Motuzas J., Martens W., Diniz da Costa J. C. (2018). Ceram. Int..

[cit13] Chen H., Motuzas J., Martens W., Diniz da Costa J. C. (2018). Appl. Catal., B.

[cit14] Zhang X., Qin J., Xue Y., Yu P., Zhang B., Wang L., Liu R. (2014). Sci. Rep..

[cit15] McLaren A., Valdes-Solis T., Li G., Tsang S. C. (2009). J. Am. Chem. Soc..

[cit16] Sun M., Jiang Y., Li F., Xia M., Xue B., Liu D. (2010). Mater. Trans..

[cit17] Leiw M. Y., Guai G. H., Wang X., Tse M. S., Ng C. M., Tan O. K. (2013). J. Hazard. Mater..

[cit18] Tummino M. L., Laurenti E., Deganello F., Bianco Prevot A., Magnacca G. (2017). Appl. Catal., B.

[cit19] Zhang L., Nie Y., Hu C., Qu J. (2012). Appl. Catal., B.

[cit20] Leo A., Motuzas J., Yacou C., Liu S., Serra J., Navarrete Algaba L., Drennan J., Julbe A., Costa J. (2017). J. Membr. Sci..

[cit21] Taran O. P., Ayusheev A. B., Ogorodnikova O. L., Prosvirin I. P., Isupova L. A., Parmon V. N. (2016). Appl. Catal., B.

[cit22] Ma J., Wang K., Li L., Zhang T., Kong Y., Komarneni S. (2015). Ceram. Int..

[cit23] Ma J., Ding J., Li L., Zou J., Kong Y., Komarneni S. (2015). Ceram. Int..

[cit24] Patil M. R., Shrivastava V. S. (2015). Appl. Nanosci..

[cit25] Kim K. N., Jung H.-R., Lee W.-J. (2016). J. Photochem. Photobiol., A.

[cit26] Liu F., Xie Y., Yu C., Liu X., Dai Y., Liu L., Ling Y. (2015). RSC Adv..

[cit27] Sathishkumar P., Mangalaraja R., Rozas O., Vergara Rosales C., Mansilla H., Pinilla M. Á., Sambandam A. (2016). Chemosphere.

[cit28] Yu T., Shen Z. X., Shi Y., Ding J. (2002). J. Phys.: Condens. Matter.

[cit29] Wang Z., Schiferl D., Zhao Y., O'Neill H. S. C. (2003). J. Phys. Chem. Solids.

[cit30] Pollak F. H. , in Semiconductors and Semimetals, ed. T. P. Pearsall, Elsevier, 1990, vol. 32, pp. 17–53

[cit31] Tatarchuk T., Bououdina M., Macyk W., Shyichuk O., Paliychuk N., Yaremiy I., Al-Najar B., Pacia M. (2017). Nanoscale Res. Lett..

[cit32] Molla A., Sahu M., Hussain S. (2015). J. Mater. Chem. A.

[cit33] Sathishkumar P., Mangalaraja R. V., Anandan S. (2016). Renewable Sustainable Energy Rev..

